# Supplementary material for: β-Amyloid as a new target to suppress tonic PTH hypersecretion in primary hyperparathyroidism
Source: medRxiv. 2025 May 28:2025.05.27.25328314. Preprint. [Version 1] doi: 10.1101/2025.05.27.25328314 (PMC12148266; doi:10.1101/2025.05.27.25328314)
Supplement: Supplement 1 [file NIHPP2025.05.27.25328314v1-supplement-1.pdf]

medRxiv preprint doi: https://doi.org/10.1101/2025.05.27.25328314; this version posted May 28, 2025. The copyright holder for this preprint (which was not certified by peer review) is the author/funder, who has granted medRxiv a license to display the preprint in perpetuity. This article is a US Government work. It is not subject to copyright under 17 USC 105 and is also made available for use under a CC0 license.

Supplementary Materials

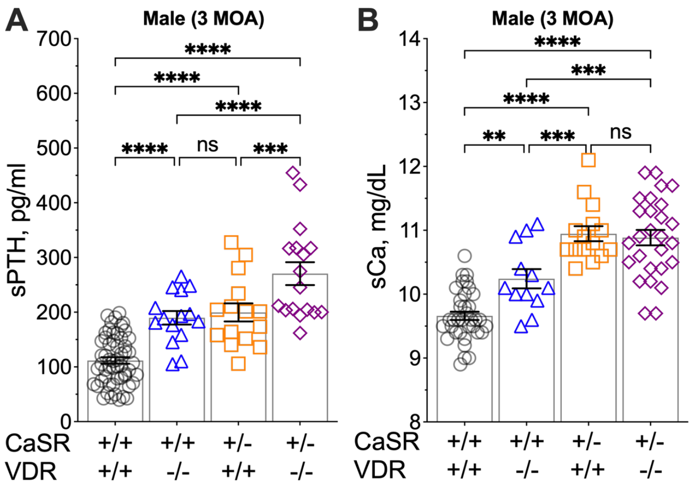

fig. S1. **Hormonal and mineral status in mouse models of HPT.** (**A**) Serum PTH (sPTH) and (**B**) total serum Ca^{2+} (sCa) of male ^{PTC}*Vdr*$^{-/-}$ (blue triangles), ^{PTC}*Casr*$^{+/-}$ (orange squares), ^{PTC}*Vdr*$^{-/-}$ *Casr*$^{+/-}$ (purple diamonds) mice and control littermates (Cont, black circles) at 3 MOA. Mean ± s.e.m. n = 10-32 mice. **$p < 0.01$, ***$p < 0.005$, ****$p < 0.0001$, and no significance (ns, $p > 0.05$) was determined by one-way ANOVA with Fisher's LSD test using Prism 10 statistics software.

medRxiv preprint doi: https://doi.org/10.1101/2025.05.27.25328314; this version posted May 28, 2025. The copyright holder for this preprint
(which was not certified by peer review) is the author/funder, who has granted medRxiv a license to display the preprint in perpetuity.
This article is a US Government work. It is not subject to copyright under 17 USC 105 and is also made available
for use under a CC0 license.

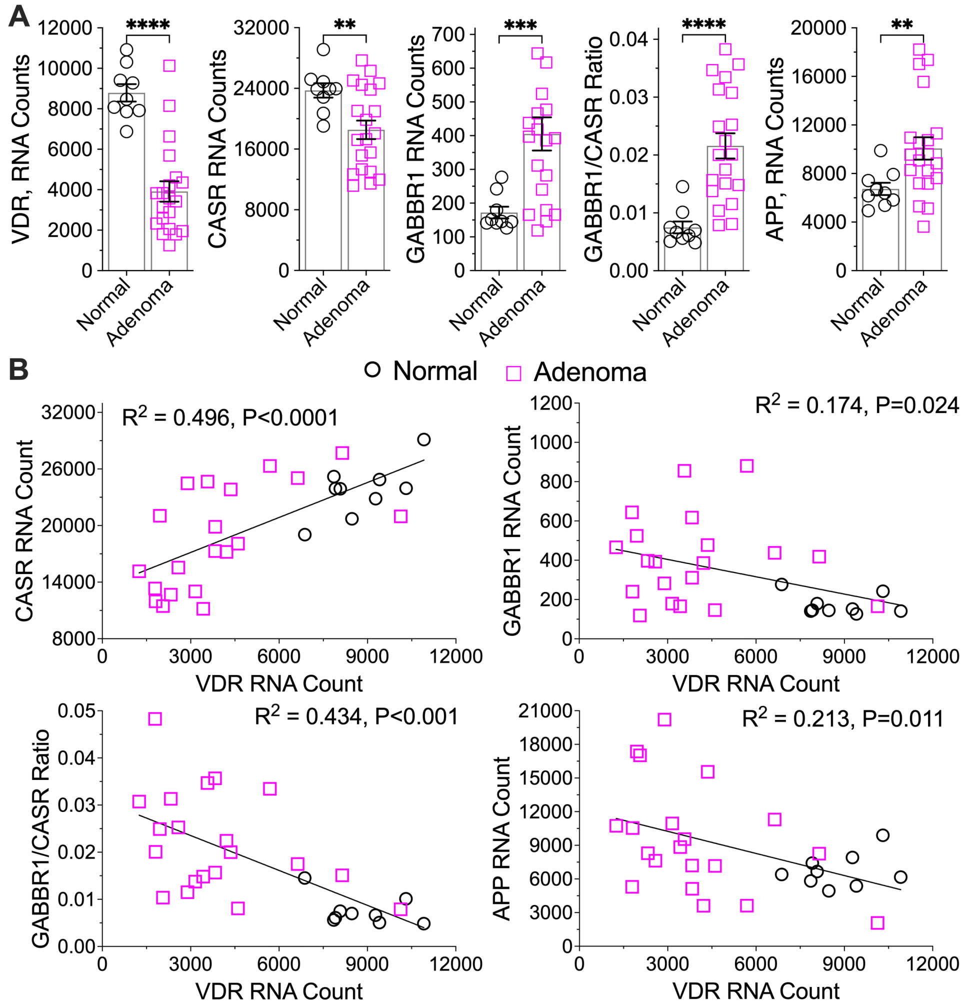

fig. S2. Decreased VDR RNA expression correlates with increased propensity of GABBR1/CaSR heterodimerization and APP expression in parathyroid adenomas from PHPT patients. (**A**) RNA abundance of VDR, CaSR, GABBR1 and APP in human parathyroid adenomas (pink squares, n = 17) and PTGs of normal donors (black circles, n = 7) was determined by NanoString nCounter gene expression assays. mean ± s.e.m. **$p < 0.01$, ***$p < 0.005$, ****$p < 0.0001$ by two-tailed student t-tests. (**B**) Correlation of each RNA level with VDR RNA level in parathyroid adenoma (pink squares) and normal PTGs (black circles) was performed by a linear regression model with derived Pearson coefficients (R^2) and two-tailed p values at 95% confidence using Prism 10 statistics software.

medRxiv preprint doi: https://doi.org/10.1101/2025.05.27.25328314; this version posted May 28, 2025. The copyright holder for this preprint (which was not certified by peer review) is the author/funder, who has granted medRxiv a license to display the preprint in perpetuity.
This article is a US Government work. It is not subject to copyright under 17 USC 105 and is also made available for use under a CC0 license.

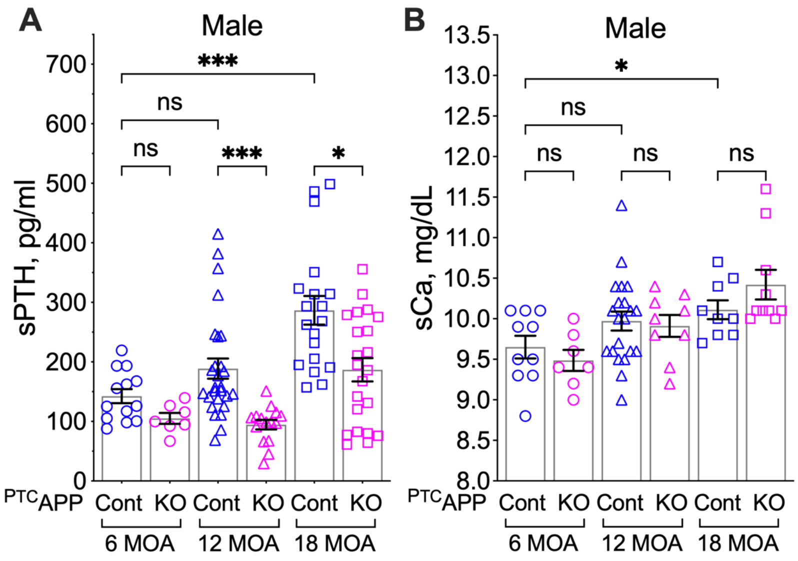

fig. S3. Impacts of PTC-specific *App* KO on hormonal and mineral status. (**A**) sPTH and (**B**) sCa levels of male $^{PTC}App^{-/-}$ (KO, pink symbols) and $App^{fl/fl}$ control (Cont, blue symbols) mice at 6 (circle), 12 (triangle), and 18 (square) MOA. Mean ± s.e.m. n = 7-26. ns ($p > 0.05$), *$p < 0.05$, ***$p < 0.005$ vs control (two-way ANOVA with Sidak's multiple comparisons test).

medRxiv preprint doi: https://doi.org/10.1101/2025.05.27.25328314; this version posted May 28, 2025. The copyright holder for this preprint (which was not certified by peer review) is the author/funder, who has granted medRxiv a license to display the preprint in perpetuity. This article is a US Government work. It is not subject to copyright under 17 USC 105 and is also made available for use under a CC0 license.

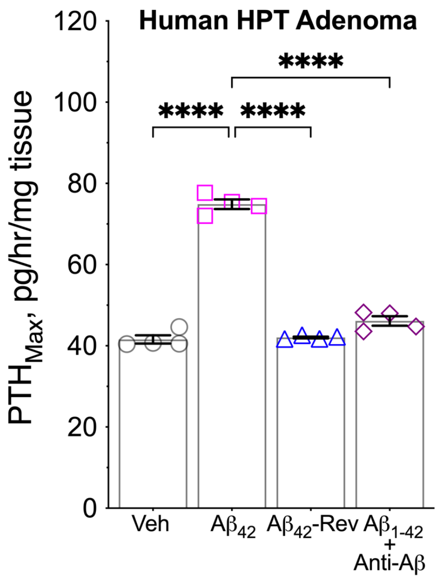

fig. S4. Effects of Aβ$_{42}$ on PTH secretion in human parathyroid adenomas from HPT patients. Resected and sectioned parathyroid adenomas were incubated with vehicle (0.1% DMSO), Aβ$_{42\text{-}1}$ (Aβ$_{42}$-Rev, 200 nM), and Aβ$_{1\text{-}42}$ (200 nM) with or without co-incubation with Aβ -neutralizing monoclonal antibody, Aducanumab (Anti-Aβ$_{42}$, 20 µg/mL). Changes in maximal PTH secretion rate (PTH$_{Max}$) on a per mg of tissue and per hour basis were determined from Ca^{2+}-response curves. mean ± s.e.m., $n = 4$ gland sections for each treatment. ****$p < 0.001$ (one-way ANOVA with Fisher's LSD test).

medRxiv preprint doi: https://doi.org/10.1101/2025.05.27.25328314; this version posted May 28, 2025. The copyright holder for this preprint (which was not certified by peer review) is the author/funder, who has granted medRxiv a license to display the preprint in perpetuity. This article is a US Government work. It is not subject to copyright under 17 USC 105 and is also made available for use under a CC0 license.

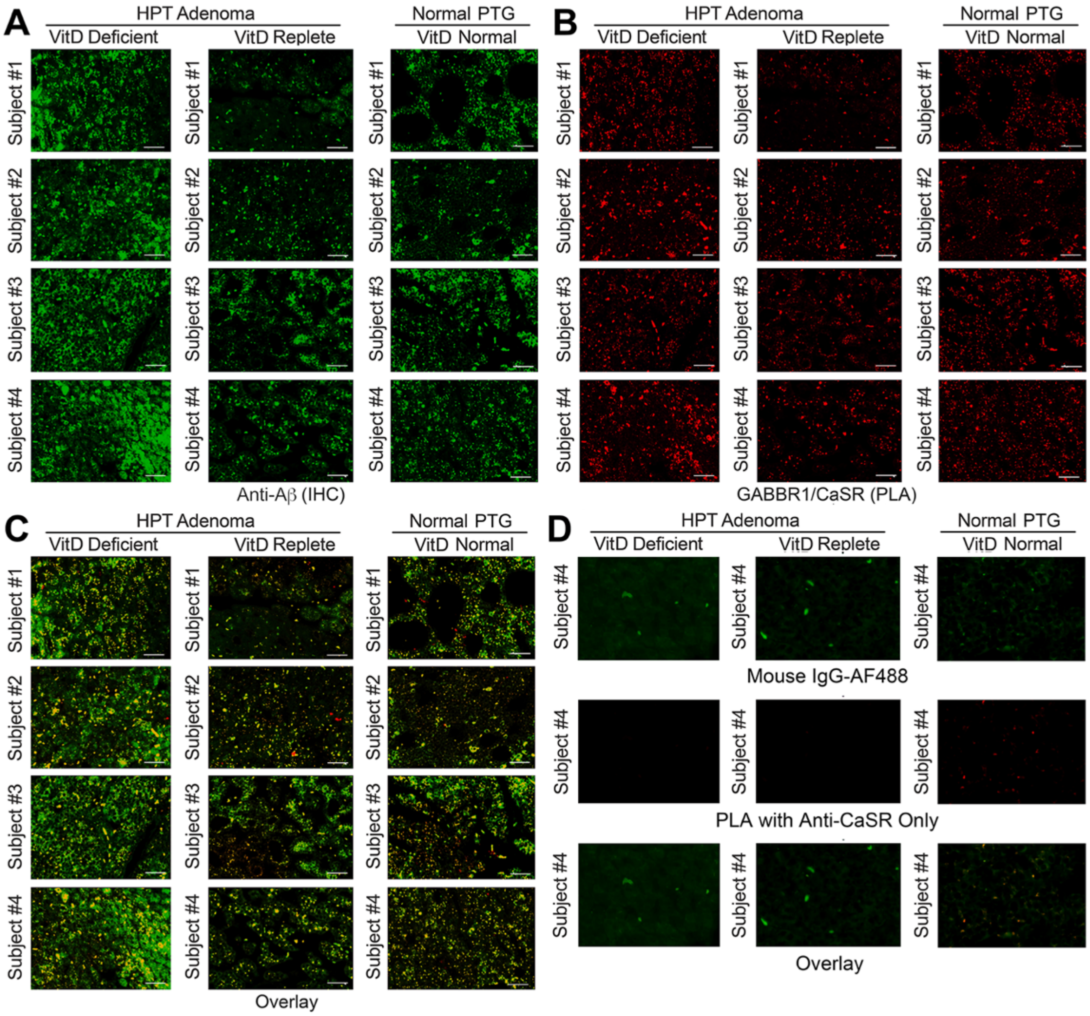

fig. S5. Vitamin D deficiency increases the expression and colocalization of Aβ_{42} with GABBR1/CaSR heterodimers. PTGs from 4 human subjects in each group of vitamin D deficient or repleted PHPT patients and normal donors were sequentially subjected to the proximity ligation assay (PLA) and immunohistochemistry (IHC) to detect endogenous GABBR1/CaSR heterodimers and Aβ_{42}, respectively. (**A**) Aβ_{42} immunoreactivity was visualized with Alex Fluor 488 (green) and (**B**) GABBABR1/CaSR heterodimer with Texas Red (red) signals. (**C**) Colocalization of Aβ_{42} and GABBR1/CaSR were visualized by yellow signals in overlayed images. Scale bar: 125 μm. (**D**) The absence of fluorescent signals of receptor heteromerization or Aβ_{42} in PTGs treated with anti-CaSR alone in PLA or control IgG in IHC respectively.

medRxiv preprint doi: https://doi.org/10.1101/2025.05.27.25328314; this version posted May 28, 2025. The copyright holder for this preprint (which was not certified by peer review) is the author/funder, who has granted medRxiv a license to display the preprint in perpetuity. This article is a US Government work. It is not subject to copyright under 17 USC 105 and is also made available for use under a CC0 license.

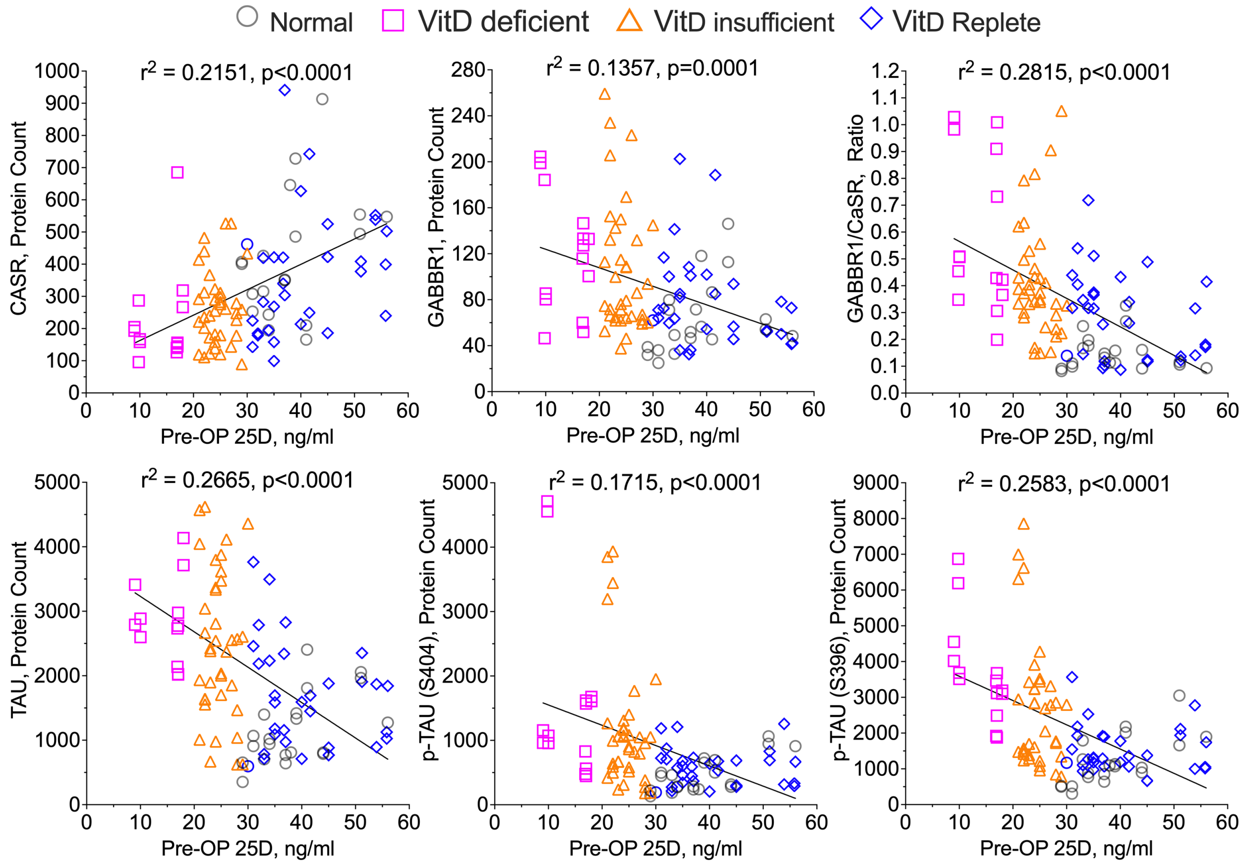

fig. S6. Pre-operative vitamin D levels inversely correlate with the propensity of GABBR1/CaSR heterodimerization and the expression and phosphorylation of Tau in human parathyroid. Protein expression in normal PTGs (grey circles) and parathyroid adenomas from PHPT patients with deficient (≤20 ng/ml, pink squares), insufficient (between 20-30 ng/ml, orange triangles), or replete (≥30 ng/ml, blue diamonds) pre-operative 25OH vitamin D levels were quantified by the NanoString GeoMx platform and correlated with pre-operative 25OH vitamin D levels using a linear regression model to derive the Pearson coefficients (R^2) and two-tailed *p* values at 95% confidence using Prism 10 statistics software.

medRxiv preprint doi: https://doi.org/10.1101/2025.05.27.25328314; this version posted May 28, 2025. The copyright holder for this preprint (which was not certified by peer review) is the author/funder, who has granted medRxiv a license to display the preprint in perpetuity.
This article is a US Government work. It is not subject to copyright under 17 USC 105 and is also made available for use under a CC0 license.

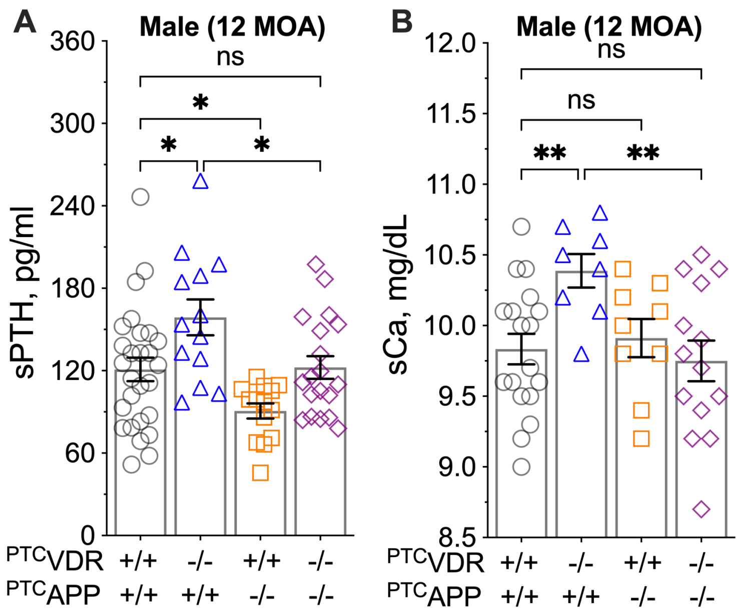

fig. S7. Impacts of PTC-specific *App* KO on hormonal and mineral status in mouse model of vitamin D deficiency. (**A**) sPTH and (**B**) sCa of male $^{PTC}Vdr^{-/-}$ (blue triangles), $^{PTC}App^{-/-}$ (orange squares), $^{PTC}Vdr^{-/-}App^{-/-}$ (purple diamonds) mice, and control littermates ($Vdr^{+/+}App^{+/+}$, grey circles) at 12 MOA. mean ± s.e.m., n = 8-28 mice for each genotype. ns ($p > 0.05$), *$p < 0.05$, **$p < 0.01$ (one-way ANOVA with Fisher's LSD test).

medRxiv preprint doi: https://doi.org/10.1101/2025.05.27.25328314; this version posted May 28, 2025. The copyright holder for this preprint (which was not certified by peer review) is the author/funder, who has granted medRxiv a license to display the preprint in perpetuity. This article is a US Government work. It is not subject to copyright under 17 USC 105 and is also made available for use under a CC0 license.

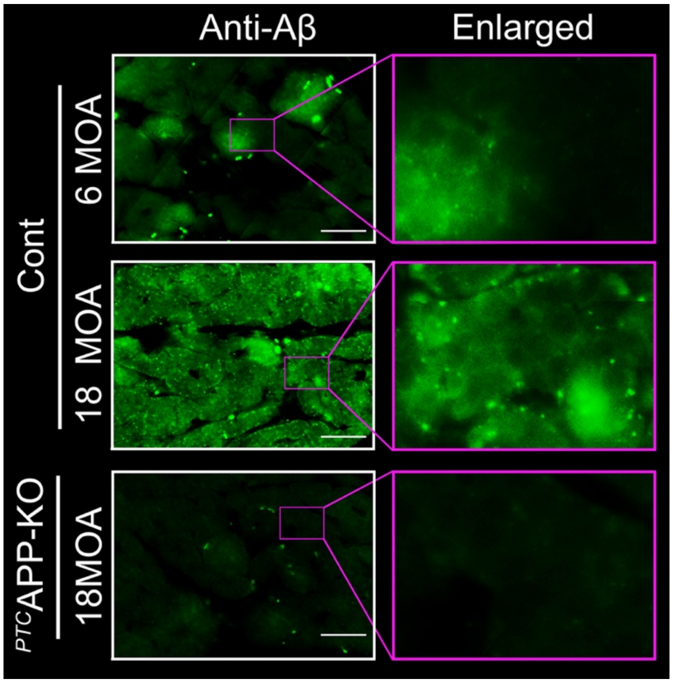

fig. S8. Increased expression of Aβ_{42} in PTGs of ageing mice. Immunohistochemical detections of endogenous Aβ_{42} in PTGs from 18 MOA ^{PTC}*App*$^{-/-}$ mice and *App*$^{fl/fl}$ control littermates at 6 and 18 MOA. Aβ_{42} immunoreactivity was visualized with Alex Fluor 488 (green). Scale bar: 125 µm.

medRxiv preprint doi: https://doi.org/10.1101/2025.05.27.25328314; this version posted May 28, 2025. The copyright holder for this preprint (which was not certified by peer review) is the author/funder, who has granted medRxiv a license to display the preprint in perpetuity. This article is a US Government work. It is not subject to copyright under 17 USC 105 and is also made available for use under a CC0 license.

Normal Donors						HPT: VitD Deficient					
Sample ID	Sex	Age (years)	Pre-op 25D (ng/ml)	Pre-op PTH (pg/ml)	Pre-op sCa (mg/dL)	Sample ID	Sex	Age (years)	Pre-op 25D (ng/ml)	Pre-op PTH (pg/ml)	Pre-op sCa (mg/dL)
AHGP057RL	Female	29-74	41	ND	10.4	PTAS0020	Female	40-79	17	100	10.7
AHBE267			39		9.1	PTAS0064			18	45	10.8
AHGY385LL			51		8.2	PTAS0104			9	119	10.9
AHGN409RU			44		9.6	PTAS0126			10	260	10.7
AGKC257			37		8.8	PTAS0141			17	220	11.3
AHHA246LU	Male		55		10.2	PTAS0094			17	62	10
AHHA004LU			38		10.1	PTAS0128			18	88	10.2
						PTAS0057			9.8	300	10.9
**Mean**		**44.6**	**43.6**		**9.5**	**Mean**		**59.1**	**14.5**	**149.3**	**10.7**
N		7	7		7	N		8	8	8	8
SD		15.6	6.9		0.82	SD		13.57	4.07	96.78	0.41
						p value vs. Normal		7.91E-02	3.14E-06	ND	7.01E-03

HPT: VitD Insufficient						HPT: VitD Replete					
Sample ID	Sex	Age (years)	Pre-op 25D (ng/ml)	Pre-op PTH (pg/ml)	Pre-op sCa (mg/dL)	Sample ID	Sex	Age (years)	Pre-op 25D (ng/ml)	Pre-op PTH (pg/ml)	Pre-op sCa (mg/dL)
PTAS0138	Female	37-80	21	112	10.7	PTAS0084	Female	38-81	30	192	10.3
PTAS0099			29	79	10.8	PTAS0125			30	69	10.4
PTAS0037			22	94	10.9	PTAS0054			31	90	9.8
PTAS0046			22	118	10.7	PTAS0025			32	157	10.4
PTAS0068			22	148	11	PTAS0136			33	74	11
PTAS0124			22	65	10.9	PTAS0059			35	147	10.4
PTAS0123			23	104	9.9	PTAS0117			35	135	11.9
PTAS0119			24	115	11.1	PTAS0086			37	115	10.9
PTAS0150			24	191	10.8	PTAS0200			37	150	9.9
PTAS0078			25	236	10.9	PTAS0140			42	125	11.2
PTAS0121			25	130	11.1	PTAS0079			45	87	10
PTAS0051			26	62	11	PTAS0093			51	66	11.1
PTAS0060			26	133	10.4	PTAS0166			56	93	10.4
PTAS0146			26	202	10.6	PTAS0115			58	120	10.6
PTAS0007			28	93	10.9						
PTAS0231			21	49	10.8						
PTAS0142			29	136	10.8						
PTAS0133			30	146	11.2						
**Mean**		**62.6**	**24.7**	**122.9**	**10.8**	**Mean**		**64.5**	**39.4**	**115.7**	**10.6**
N		18	18	18	18	N		14	14	14	14
SD		10.68	2.89	49.65	0.30	SD		12.27	9.56	37.78	0.57
p value vs. Normal		2.17E-02	2.44E-04	#DIV/0!	4.75E-03	p value vs. Normal		3.71E-01	6.58E-08	3.74E-01	6.60E-01

table S1. **Demographics and serum chemistry of HPT patients and normal PTG donors.** Vitamin D deficiency, insufficiency, and replete status are defined as pre-operative 25OHD levels ≤ 20 ng/ml, between 20- 30 ng/ml, and ≥ 30 ng/ml, respectively, based on Institute of Medicine Guidelines (*34*). Mean ± s.e.m. *p*-value versus normal donors were determined by two-tailed Student's t-test are indicated.
